# The Regulatory-T-Cell Memory Phenotype: What We Know

**DOI:** 10.3390/cells11101687

**Published:** 2022-05-19

**Authors:** Julia N. Khantakova, Aleksey S. Bulygin, Sergey V. Sennikov

**Affiliations:** Research Institute of Fundamental and Clinical Immunology (RIFCI), 630099 Novosibirsk, Russia; aleksej.bulygin95@mail.ru (A.S.B.); sennikov_sv@mail.ru (S.V.S.)

**Keywords:** regulatory T-cell memory, Treg cells, immune memory, flow cytometry, cell markers

## Abstract

In immunology, the discovery of regulatory T (Treg) cells was a major breakthrough. Treg cells play a key role in pregnancy maintenance, in the prevention of autoimmune responses, and in the control of all immune responses, including responses to self cells, cancer, infection, and a transplant. It is currently unclear whether Treg cells are capable of long-term memory of an encounter with an antigen. Although the term “immunological memory” usually means an enhanced ability to protect the body from reinfection, the memory of the suppressive activity of Treg cells helps to avoid the state of generalized immunosuppression that may result from the second activation of the immune system. In this review, we would like to discuss the concept of regulatory memory and in which tissues memory Treg cells can perform their functions.

## 1. Introduction

One of the key features of adaptive immunity is the capacity to efficiently and more rapidly respond to a previously encountered antigen. Two areas in immunological memory have been studied the most: humoral immunity (which includes antibodies, memory B cells, and plasma cells) and cellular immunity (which involves memory CD8^+^ and CD4^+^ T cells) [1]. It is in the acute phase of infection that activated effector T cells are selected, as are future memory T cells [2]. The latter cells in a certain amount persist in the body for decades, ready to mediate an enhanced and accelerated response to reinfection. Furthermore, aside from being a source of new effector T cells, some memory T cells can strengthen memory B-cell responses and antibody production after reinfection. Memory cells evade apoptosis and persist in various parts of the body for a long time [3]. Specific features of memory T-cell subsets are primarily their phenotype, migratory properties, and tissue-homing patterns, which in many cases imply unique functional attributes [4]. Expression of markers and transcription factors as well as epigenetic landscapes and metabolic profiles have been investigated especially well in memory CD8^+^ T cells [5]. Although a lot is known about the effector function of T helper cells, the role and biology of memory CD4^+^ T cells are more complicated and less understood [4]. Moreover, the function of memory CD4^+^ T cells in the development of an immune response is only partially determined by the subset of T helper progenitor cells from which a primary immune response arises [6]. An additional layer of complexity is various subpopulations of memory CD4^+^ T cells generated in the course of a primary immune response [7,8].

Because of the common origin of T helper cells and regulatory T (Treg) cells (Box 1), it seems logical that the formation of memory Treg cells (mTregs) proceeds in parallel with the development of effector memory T cells. On the other hand, can long-term persistence of suppressive cells after the resolution of an initial antigenic insult lead to (i) the suppression of effector responses of memory T cells after a second encounter with the antigen and (ii) to persistent immunosuppression? Recently, the suppressor function of antigen-specific Treg cells was found to be a transient property [9]. In that experiment, after resolution of an inflammatory process, Treg cells reversed their activation-specific transcriptional changes and decreased their own suppressive function over time. In addition, several studies have shown that cues in a microenvironment, e.g., stress, danger, or inflammation, can abrogate forkhead box P3 (Foxp3) expression and launch the production of effector cytokines, resulting in so-called ex-Foxp3 cells [10]. Thus, does a long-lived population of mTreg cells exist? In this review, we would like to discuss the concept of regulatory memory and in which tissues mTreg cells can perform their functions.

Box 1 **Regulatory T cells.**

Regulatory T cells (Tregs) are a specialized subset of immunosuppressive CD4^+^ T cells that express lineage-specific transcription factor forkhead box P3 (Foxp3) [11,12]. Treg cells ensure immunological homeostasis by substantially suppressing autoreactive CD4^+^ T cells that have escaped negative selection in the thymus [13]. Besides, Treg cells act as key negative regulators of inflammation in various pathological conditions, including infections, autoimmune diseases, and cancer [14,15,16,17]. Treg cells drastically increase their suppressive function in response to inflammation. Activated Treg cells raise the levels of immunosuppressive proteins (IL-10 and/or TGF-β) and chemokines, and they undergo polycomb-mediated repression of Foxp3-bound genes; this process may prevent the acquisition of proinflammatory functions [12]. Treg cells that infiltrate wounds express epidermal growth factor receptor (EGFR) and take part in tissue regeneration. A conditional knockout of EGFR in Treg cells delays wound closure and enhances the accumulation of proinflammatory macrophages [18].

## 2. The Origin of Treg Cells

**Figure 1:**
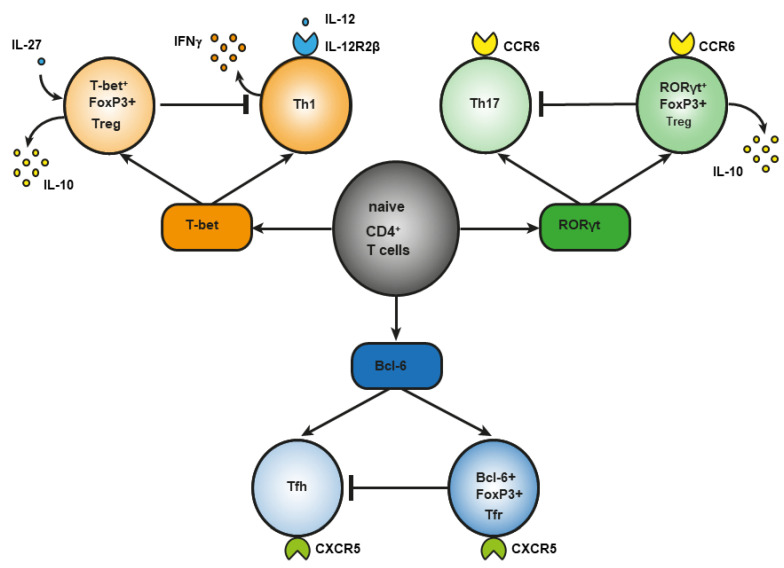
Differences between effector CD4^+^ T lymphocytes and Treg cells in the role of a master regulator in their differentiation.

During infection, under the influence of signals from a Toll-like receptor (TLR) [19] of proinflammatory cytokines [20] or of costimulation through CD40 [21], dendritic cells are activated, an effector response is triggered, and induced Treg cells develop [22]. This notion is supported by the involvement of the same transcription factors in the differentiation of phenotypically similar subpopulations of CD4^+^ effector T cells and Treg cells, although there are key differences. For example, transcription factor T-bet is required for the differentiation of T helper 1 (Th1) cells and the differentiation of T-bet^+^ Treg cells (Figure 1). In response to type I interferon (IFN), IFN-γ or interleukin-27 (IL-27), STAT 1 (signal transducer and an activator of transcription 1) are phosphorylated, and T-bet expression is triggered. T-bet in turn induces the expression of IL-12Rβ2 on the cell surface and enhances the sensitivity of Th1 cells, not of Treg cells, to IL-12 [23,24]. IL-12 activates STAT4 resulting in the T-bet upregulation required for full differentiation of Th1 cells. In contrast to Th1 lymphocytes, the activation through T-bet in Treg cells does not elevate IL-12Rβ2 expression and IFN-γ production [23]. Therefore, the sensitivity of Treg cells to IL-12–dependent differentiation does not increase owing to the presence of inhibitory tri-methyl histone marks (H3K27) in the *Il12rb2* promoter [23]. Nevertheless, after prolonged stimulation with IL-12, these Treg cells lose their suppressive function and begin to produce a large amount of IFN-γ [25]. In type 1 diabetes mellitus and in multiple sclerosis, an elevated number of Treg cells secreting IFN-γ is associated with disease aggravation. This observation suggests that reprogrammed Treg cells may contribute to autoimmunity pathogenesis [26,27].

Regulatory T (Treg) cells constitute a specific anti-inflammatory lineage of CD4^+^ T-lymphocyte differentiation determined by X-linked transcription factor Foxp3 [28]. Nonetheless, some activated Treg cells can express transcription factors (T-bet and RORγt) characteristic of effector CD4^+^ T cells [24,29,30,31]. In a steady state, activated Treg cells temporarily upregulate T-bet for their own homeostasis [31]. Among tumor-infiltrating Tregs, investigators detected T-bet^+^Foxp3^+^CD4^+^ T cells with a higher expression of typical Treg factors, such as ICOS, GITR, CD103, CTLA4, PD-1, and IL-10 as compared to CD4^+^ T cells, which express either T-bet or Foxp3 [32]. T-bet–deficient Treg cells are characterized by reduced survival and by a failure to inhibit Th1-mediated inflammation after adoptive transfer into Scofy mice [33]. On the other hand, there are several studies indicating that T-bet deficiency does not affect the suppressive function of Tregs [34,35]. In the small-intestine lamina propria, a population of resident RORγt^+^FoxP3^+^ Treg cells was found that has an activated phenotype (CD44^high^CD62L^low^) and strongly expresses typical Treg factors ICOS and CTLA-4 and nucleotidases CD39 and CD73 [29]. The presence of RORγt^+^FoxP3^+^ Treg cells is crucial, and it reduces the risk of colitis and colorectal cancer [36]. There are fewer RORγt^+^ Tregs in the gut of patients with food allergy, and commensal-bacteria–mediated protection from food allergy depends on RORγ^+^ Tregs [37]. Resident RORγ^+^ Tregs and Th17 lymphocytes reduce the severity of M. tuberculosis–driven lung inflammation in mice [38]. Bcl-6^+^FoxP3^+^ Treg cells strongly express coinhibitory protein PD-1. In mice, B cell lymphoma (Bcl) 6 deficiency in Foxp3^+^ Tregs aggravates experimental Sjögren’s syndrome, but at the same time it enhances immunity to an influenza virus [39]. In breast cancer, Bcl+FoxP3+ Treg cells promote the formation of the B cells that produce IL-10 [40]. Abbreviation: bcl-6, B cell lymphoma (Bcl) 6; CCR, C-C chemokine receptor; CTLA-4, cytotoxic T-lymphocyte-associated protein; CXCR, CXC chemokine receptor; FoxP3, forkhead box; GITR, glucocorticoid-induced tumor necrosis factor-related receptor; ICOS, inducible costimulator; IL, interleukin; PD-1, programmed cell death protein 1; RORγt, RAR-related orphan receptor gamma; Tfh, T follicular helper cells; Tfr, T follicular regulatory cells; Treg cells, regulatory T-cells.

Another example of differences in the role of a master regulator of T-cell differentiation is RAR-related orphan receptor gamma (RORγt). It is known that RORγt is important for the differentiation of Th17 lymphocytes and for the expression of C-C chemokine receptor (CCR) 6 [41]. The signaling initiated by cytokines of the IL-17 family via STAT3 is known to cause the differentiation of Th17 lymphocytes [42]. On the other hand, in the lamina propria of the small intestine in humans and mice, a population of Treg cells has been found that also expresses RORγt [43,44] and CCR6 [45]. The production of such RORγt^+^ Tregs involves STAT3-dependent transcriptional pathways (similar to those in Th17 lymphocytes), and it requires microbiota antigens for their differentiation [44,46]. Loss of STAT3 leads to downregulation of CCR6 in RORγt^+^ Tregs, and it impairs their migration to the intestine [47]. In Th17 cells, IL-6 is required for the activation of a factor called STAT3, whereas IL-10 is required for this process in Treg cells [47]. CCR6 may direct the migration of Treg cells to sites of Th17-mediated inflammation, suggesting that these CCR6^+^ Treg cells may be especially potent suppressors of Th17 responses [48]. Mice that are deficient in RORγt^+^FoxP3^+^ Tregs develop a severer and more lethal type of oxazolone-induced colitis, which is a model of ulcerative colitis [36,49,50]. Nevertheless, in the presence of IL-1, IL-23, IL-6, and transforming growth factor beta (TGF-β), naïve CD25^+^FoxP3^+^ Treg cells show an upregulation of RORγt—along with downregulation of FoxP3 and a loss of suppressor functions—and they differentiate into Th17 lymphocytes [51].

Bcl-6^+^ “T follicular regulatory” (Tfr) cells express B-cell–associated CXC chemokine receptor (CXCR)5 and develop in parallel with Bcl-6^+^ T follicular helper (Tfh) cells, which contribute to humoral immunity [52,53]. Tfr cells also express a Bcl-6 antagonist called Blimp-1, which is not expressed by Tfh cells [54]. Transcription factor NFAT2 is required for CXCR5 expression in Tfr cells but not in Tfh cells [55], thus further supporting the notion that effector T cells and Tregs use different molecular pathways to attain similar phenotypes. The main function of Tfr cells is thought to be the suppression of the germinal cancer reaction and the inhibition of B-cell proliferation and immunoglobulin production [40].

Accordingly, it is obvious that Treg cells are a heterogeneous population. Depending on their origin, Tregs are categorized into two subpopulations: natural (nTregs) and induced Tregs, i.e., those formed in secondary lymphoid organs during an immune response (iTregs) [11,13,56]. In mice, transcription factor Helios was identified as a marker discriminating between thymic Treg cells (Helios^+^) and peripheral Treg (pTreg) cells (Helios^−^) [57]. In the present review, we assume that nTreg cells are (i) Tregs that arise in the thymus during negative selection of CD4^+^ T lymphocytes (tTregs) [12] and (ii) Tregs originating in the periphery when stimulated by the commensal microbiota (pTregs) [58,59]. According to the literature, >90% of Treg cells of adipose tissue [60], ~70–80% of Treg cells of the intestine in newborn mice, and ~30% of Treg cells in the intestine of adult animals [61] are of thymic origin, as are Treg cells of the skin [62]. There is a hypothesis that such pTreg cells of barrier tissues constitute a major proportion of mTreg cells and are similar in function to tissue-resident memory T cells [59,62,63]. This theory is supported by evidence that pTreg lymphocytes in the gut and in the skin are necessary to suppress immune responses against local commensal bacteria [62,64,65,66].

A number of subpopulations of Treg cells can be distinguished depending on the phenotypic profile, too. First of all, there are CD4^+^ Treg cells and CD8^+^ Treg cells [67,68,69,70]. Second, depending on the expression of CD25, FoxP3, CD127, CD39, CD45RA, CTLA4, GITR, Helios, ICOS, PD-1, FasL, and perforin as well as the secretion of cytokines IL-10, IL-35, and TGF-β, Treg cells can be further categorized into subpopulations that have been described previously, and their description is outside the scope of this review [71]. A distinctive feature of Treg cells is the expression of the transcription factor FoxP3 [56], which is required for the establishment and the maintenance of their suppressor activity [10]. Loss of Foxp3 expression leads to the development of a lethal multiorgan autoimmune disease in mice and humans [72,73]. On the other hand, there are FoxP3^−^ Treg cells: type 1 regulatory cells, and IL-35-Producing T Cells (iTR35). They do not express FoxP3 but produce the immunosuppressive cytokine IL-10 [74], and iTR35 cells also secrete IL-35 [75].

It is possible that similarly to subpopulations of CD4^+^ T cells, Treg cells should be categorized not by phenotypic profiles [76,77] but rather by participation in the suppression of certain effector functions. Depending on microenvironmental factors, differentiating CD4^+^ Treg cells acquire either suppressor or effector potential. This approach may facilitate the search for specific mTreg markers, which are still being discussed [78].

## 3. Immunologic Memory

In 1978, researchers isolated a long-lived population of antigen-specific suppressor cells from T-cell compartments [79]. Under conditions of adoptive transfer after a second encounter with an antigen, in that study, these cells deployed their suppressor activity severalfold faster. That work is thought to be the first to formulate the concept of regulatory memory. Thanks to modern technical advances, it is now feasible to perform a more accurate analysis of regulatory cell populations.

It is known that effector memory cells form only in response to an antigen. Nonetheless, their survival does not depend on the antigen’s further presence in the microenvironment. Although the antigen-specific nature of Treg-cell differentiation has already been confirmed by many reports [80,81], the mechanisms underlying long-term survival of Treg cells in the absence of an antigen have not yet been sufficiently investigated. T-cell receptor (TCR) of tTreg cells is specific for autoantigens that are always present in the body. Taking this into account, an additional difficulty is the identification of mTreg cells having non-self specificity, i.e., not specific to innate antigens.

Another feature of memory cells is a faster and more potent reaction to a second encounter with a pathogen. In effector cells, scientists measure the proliferation rate, cytokine production, and pathogen elimination kinetics, but these criteria are not suitable for mTreg cells. Treg cells secrete a limited repertoire of cytokines, most of which are difficult to quantify per cell. In addition, the effector function of Treg cells is not always determined by cytokines. Depending on the type of Treg cells and their location, different mechanisms of tolerance induction may prevail [8,82,83].

The emergence of the CD45RO^+^ isoform instead of the CD45RA^+^ isoform is considered the main phenotypic feature of effector memory T cells [84,85,86], as is overexpression of CD44 [87,88]. Proteins CD62L and CCR7, which mediate cell homing to lymphoid organs, are now used in combination with markers CD45RA/CD45RO for phenotypic detection of subsets of memory CD8^+^ T cells [89]. Furthermore, CD47, transcription factor T-bet, LY6G, and specific epigenetic landscapes have been utilized as evidence for prior activation and/or differentiation of effector memory CD4^+^ T cells [90,91,92]. Unfortunately, most of these markers are not suitable for strict identification of mTreg cells. For instance, high expression of CD44 is necessary for Treg cells to carry out the suppressive function [93,94]. The difficulty with the search for markers may also be due to the finding that when activated, Treg cells start expressing more of already present biomolecules such as CTLA4, CD25, ICOS, and GITR, and they do not produce them de novo [95]. Lately, there are discussions about the need to rely more on quantitative shifts in the expression of markers rather than on changes in their profile [78,96]. Already known differences in the levels of marker expression among various tissues point to variation in the phenotype of mTreg cells at different sites in the body. There may be additional epigenetic markers that can indicate stable expression of FoxP3 [97] and the expression of other transcriptional regulators that participate in Treg-cell differentiation.

## 4. Skin Tissue-Resident Tregs as Memory Cells: Pros and Cons

Controlled induction of a certain antigen in the skin of mice is used as a model for studying resident Treg cells. For this purpose, transgenic mice have been created in which the expression of an antigen of interest can be induced in keratinocytes [78,98,99]. Because researchers can turn off antigen expression, they can characterize how antigen-specific memory T cells are preserved without constant exposure to the cognate antigen. Given that the expression of the model antigen in those studies was not impaired in the thymus, antigen-specific Treg cells successfully formed in the thymus and actively spread to secondary lymphoid organs. When the antigen expression was turned on, there was an activation and a proliferation of Treg cells and then their migration into the skin to, evidently, eliminate the inflammatory response. After the antigen was turned off, a population of CTLA4^hi^ Treg cells remained in the skin for a long time. When the model antigen was reintroduced, the resolution of inflammation was quicker as compared to the first time. This was the first evidence that antigen-specific FOXP3^+^ Treg cells could meet the criteria for immunological memory and persist like true memory cells.

In 2006, special skin-resident Treg cells were isolated from humans, had typical properties of effector memory cells, and were named mTreg cells [100]. In mice, it has been demonstrated that CD4^+^Foxp3^+^ T cells get into the skin on embryonic day 6–13 and stay there during the neonatal period, mediating tolerance to commensal nonpathogenic microbes; among these T cells, 80% are highly activated Treg cells [62]. Similar results have been obtained in humans: contrary to adults, in the barrier tissues of infants, Treg cells constitute 20–40% of the CD4^+^ T-cell population [63], especially in the mucosa and lymphoid tissues. In adults, Treg cells are predominantly found in lymphoid tissues and represent 2–5% of CD4^+^ T cells [63]. Tregs in the blood and lymphoid tissues have the CD45RA^+^CCR7^+^ phenotype, suggestive of a naïve state, whereas Treg cells in some regions of the mucosa have the CD45RA^−^CD69^+^CD45RO^+^ phenotype, just as conventional tissue resident T-memory cells [63]. In addition, in lymphoid organs of children, Treg cells express more FoxP3.

In human skin, according to various authors, 70% to 85% of the total pool of tissue-resident memory T cells consists of CD4^+^ T cells [4]. Among these tissue-resident memory CD4^+^ T lymphocytes, approximately 10% of cells express the transcription factor Foxp3, and they are believed to possess regulatory functions [66]. Unlike typical Treg cells, skin mTregs are completely demethylated in the *TSDR* region of the *Foxp3* gene, and they therefore exhibit stably differentiated stages [101]. Almost all Tregs in adult skin express CD45RO, whereas a substantial proportion of Tregs in fetal skin are naïve CD45RA^+^ cells [101]. mTreg cells in human skin express memory markers (CD27 and BCL-2) and elevated amounts of activation markers (CTLA4, CD25, and ICOS). Unlike mTreg cells in mouse skin, those in human skin express much less IL-2, IL-7, and its receptor CD127, but there is no difference in either IFN-γ or IL-10 expression [101]. In mice, IL-7 is necessary to maintain mTreg cells in the skin in a stable state, and IL-2 is required for the formation of mTreg cells from naïve progenitors of CD4^+^ T cells [102].

**Figure 2:**
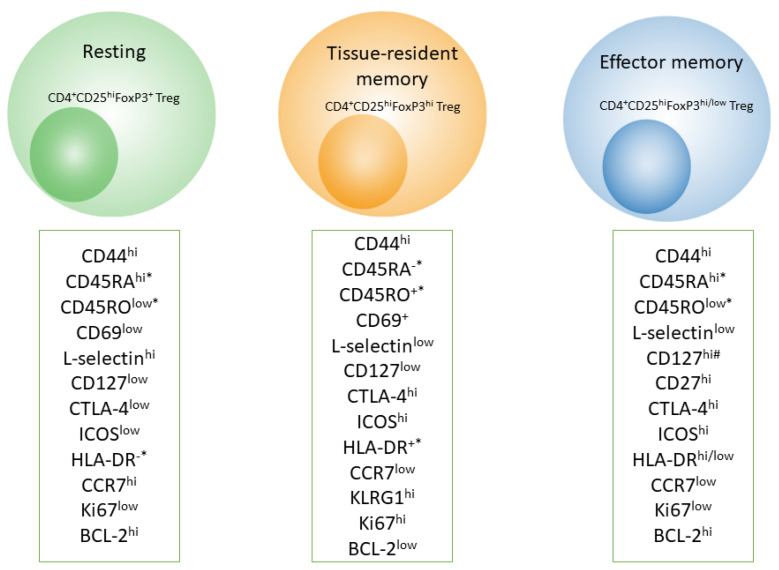
Some of markers for resting, tissue-resident and effector memory T-regulatory cells. BCL, B cell lymphoma; CCR CC-chemokine receptor; CTLA4, cytotoxic T lymphocyte antigen 4; FOXP3, forkhead box P3; ICOS, inducible T cell co-stimulator; KLRG1, killer cell lectin-like receptor subfamily G member 1; * Human only. ^#^ Shown only in mouse skin.

In contrast to skin effector memory T cells, mTreg cells have unique TCR sequences, they do not express CCR7, and they do not migrate from the skin in vivo [103]. Skin mTreg cells proliferate in an antigen-independent manner upon contact with dermal fibroblasts in the presence of IL-15 [104]. Additionally, Treg cells in adult human skin highly express other markers of T-cell memory, for example, killer cell lectin-like receptor subfamily G member 1 (KLRG1), glucocorticoid-induced tumor necrosis factor-related receptor (GITR), L-selectin (CD62L), C-C chemokine receptor (CCR) 4, CCR6, and inducible costimulator (ICOS) (Figure 2) [102]. Besides, most CD4^+^ and CD8^+^ mTreg cells found in the lamina propria of the small intestine contain low amounts of markers CD44 and CD45RB, and this feature makes them similar in phenotype to effector memory T cells [105].

These observations imply that human skin contains Treg cells that recognize unique antigens and that they are stably present in this tissue. This population may be most similar to tissue-resident or effector memory cells. A question remains whether the cells described above are two different populations of mTreg cells or one population of mTreg cells that has characteristics of resident and effector cells. Finally, the factors needed to maintain these activated mTreg cells in the skin and the specific antigens they recognize are yet to be identified.

## 5. Peripheral-Blood Treg Cells and Effector Memory

After vaccination or during acute viral infection in mice, there is an increase in the number of virus-specific Treg cells [14,16,106]. They are characterized by underexpression of CD62L and preservation of the CD44 expression level. After virus elimination, the number of Treg cells declines, but a small percentage of virus-specific Treg cells persists for more than 50 days after the infection. Upon the second administration of the Treg antigen, these cells begin to actively proliferate while strongly suppressing the growth of the effector T-cell population and reducing the production of cytokines in both systemic and tissue-specific models of reinfection [16,107]. Besides, adoptive transfer of virus-specific mTregs (in contrast to naïve Tregs) to recipient mice significantly attenuates body weight loss and lung infiltration by immune cells during influenza A virus infection [107]. According to that study, mTregs have a competitive advantage when migrating to the lungs, better control of CD4^+^ and CD8^+^ T-cell proliferation in vitro, and they suppress CD40 and CD86 activation on bone marrow dendritic cells. Furthermore, there is less tissue damage without a worsening of virus elimination. Taken together, these data support the hypothesis that mTreg cells are generated to regulate potent memory effector responses and to prevent the collateral damage to tissues that occurs with sustained immunostimulation during infection. Nevertheless, how mTreg cells reduce tissue inflammation without worsening pathogen clearance remains to be determined.

It has been demonstrated that mTregs possessing the phenotype of effector memory T cells (CD4^+^CD25^+^FoxP3^+^CD62L^lo^) can quickly migrate to nonlymphoid tissues such as the liver, lungs, and CNS (Box 2), and they can prevent tissue damage during secondary infection (Figure 2) [16]. In addition, upon subsequent viral infection, mTreg cells release a considerable amount of IL-10, thereby exerting control over the activity of CD4^+^ T lymphocytes [16].

Box 2 **Treg cells in the central nervous systems (CNS).**

Despite the immunoprivileged status of the brain, a subpopulation of regulatory T (Treg) cells has been discovered in the CNS of mice and humans. These CNS-resident Treg cells have a varied specific activation/memory phenotype, and they play an important role in the regulation of CNS inflammation. In the rat brain, Treg cells constitute ~15% of CD4^+^ T lymphocytes [108], a substantial number of these Tregs have the activation/memory phenotype (TCRαβ^+^CD4^+^Foxp3^+^CD44^+^CD62L^−^). These Tregs overexpress other markers too, including inducible T cell co-stimulator (ICOS), CD103, killer cell lectin-like receptor subfamily G member 1 (KLRG1), and cytotoxic T lymphocyte antigen 4 (CTLA4) [109]. Human cerebrospinal fluid is also dominated by T cells with the activation/memory phenotype [110]. Notably, brain astrocytes may play a regulatory part in the control of cerebral Treg cells by promoting Foxp3 expression via the IL-2–STAT5 pathway [108]. Neurons contribute to the differentiation of Treg cells as well, via B7 and the TGF-β1 pathway [109]. After intrauterine infection, there are behavioral disorders in the offspring, along with a proinflammatory T-cell immune pattern in the periphery and IL-6 upregulation in brain astrocytes [81]. Adoptive transfer of only antigen-specific Treg cells—from the dams that had toxoplasmosis during pregnancy—into 8-week-old off-spring reversed these immune and behavioral abnormalities; the injection of nonspecific Treg cells from control mice had no such effect [81]. That study confirms the presence of a long-lived population of antigen-specific Treg cells after infection and points to therapeutic potential of adoptive transfer of Treg cells in infections and in neuropsychiatric disorders comorbid with immune aberrations.

The peripheral blood of healthy people contains two phenotypically and functionally dissimilar subsets of Tregs: resting CD45RA^+^FOXP3^low^ cells and activated CD45RA^−^FOXP3^hi^ cells [111]. Both subpopulations are stable, have high suppressive activity, and do not produce effector cytokines. Human umbilical cord blood contains the largest number of resting CD45RA^+^FoxP3l^ow^ Treg cells and relatively few activated CD45RA^−^FoxP3^hi^ Treg cells [111,112]. CD45RA^+^FOXP3^low^ Treg cells readily proliferate and transform into activated CD45RA^−^FOXP3^hi^ Tregs when stimulated in vitro or in vivo [113]. It can be hypothesized that some of these activated Treg cells in the PBMC population of human blood are mTregs that persist in the absence of antigenic stimulation. Resting CD45RA^+^FOXP3^low^ Treg cells of umbilical cord blood express CD31, indicating recent emigration from the thymus [114,115,116,117]. Meanwhile, some conventional effector T cells that are recent thymic emigrants are known to undergo peripheral post-thymic proliferation giving rise to a long-lived memory T-cell population that can maintain a naïve pool of T cells in the elderly [114]. Therefore, it can be theorized that CD45RA^+^CD31^+^ Treg cells develop in a manner comparable to that of conventional effector cells. Indeed, CD45RA^+^CD31^+^ Tregs that are recent thymic emigrants have been found in the blood of women during pregnancy; these cells differentiate into CD45RA^−^CD31^−^ mTregs during normal pregnancy. On the contrary, in women with preeclampsia, CD45RA^−^CD31^+^ Tregs predominate, and the suppressor activity of CD45RA^+^CD31^+^ Tregs and CD45RA^−^CD31^−^ mTregs is low [118].

In various pathologies in humans, two different subpopulations of CD45RA^−^FOXP3^hi^ cells have been identified: effector memory Treg cells (CD25^+^CD45RA^−^CD62L^−^) and central memory Treg cells (CD25^+^CD45RA^−^CD62L^+^) overexpressing CTLA4, ICOS, and HLA-DR [119,120,121,122]. Antigen-activated CD45RA^−^FoxP3^+^ cells can also be classified into two subpopulations, depending on HLA-DR expression. HLA-DR^+^ cells have a more activated phenotype, express greater amounts of Foxp3, and have stronger suppressor activity but produce smaller amounts of effector cytokines [123,124,125]. Some authors suppose that these are mTreg cells [78]. By contrast, during normal pregnancy (Box 3), the suppressor activity of HLA-DR^+^ Treg cells diminishes as compared to nonpregnant women [126]. In the first 5–8 weeks of pregnancy, the number of naive CD45RA^+^ cells sharply drops, while the number of HLA-DR^+/−^ Tregs goes up. Subsequently, the proportion (%) of naïve CD45RA^+^ Treg cells steadily increases while the percentage of HLA-DR^+^ Treg cells goes down. Because HLA-DR expression on activated effector CD4^+^ T cells is seen during chronic infection [127], it is likely that CD45RO^−^HLA-DR^+^FoxP3^+^ Treg cells are not memory cells but represent an activated subpopulation of Tregs. On the other hand, the pool of naïve CD45RA^+^ Treg cells decreases with age in women, but the proportion of HLA-DR^+^ and HLA-DR^−^ Tregs increases [126].

Box 3 **Regulatory (Treg) cells in pregnancy.**

Pregnancy is a physiological state of a woman’s body that necessitates the induction and the maintenance of tolerance to the allogeneic fetus. During pregnancy, a systemic increase in the number of maternal Treg cells takes place, which is necessary to maintain tolerance to paternal antigens [128,129] and forms a memory for fetal antigens. In humans early in pregnancy, mTregs have been found in the peripheral blood and decidua as well [126,130,131]. A decrease in the number of Treg cells is attributed to pregnancy pathologies: preeclampsia, preterm labor, and miscarriage [130,132,133,134]. In the abortion-prone CBA/J × DBA/2 model, a higher rate of embryo resorption is observed along with simultaneous depletion of Treg cells [128]. Adoptive transfer of Treg cells from normal pregnant mice can prevent fetal rejection [135] and considerably reduce the fetal absorption rate [136,137]. In addition, promising data have been obtained about the treatment of miscarriage by means of adoptive transfer of Tregs [138]. Depletion of functional Treg cells in the third trimester can lead to preterm labor and poor neonatal outcomes, which can be reversed by adoptive transfer of Treg cells [139]. During the second pregnancy, accelerated expansion of Treg cells is due to the proliferation of fetus-specific Foxp3^+^ T cells that got preserved during the previous pregnancy [140]. The elevated number of fetus-specific Treg cells during the second and subsequent pregnancies provides better protection against disorders of fetal tolerance [140,141]. This phenomenon explains the lower number of immunological complications in subsequent pregnancies compared with the first one.

Consequently, despite the fragmentary inconsistent data, the existence of resident and/or effector memory Treg cells in barrier tissues (such as the skin and mucous membranes) and in the brain as well as in various states of the body is already becoming apparent. The discovery of tissular specialized subsets of mTreg cells affords major opportunities for targeted treatment of local inflammatory and autoimmune diseases through the activation of these cells.

## 6. Prospects of mTreg-Based Immunotherapies in Various Diseases

**Figure 3:**
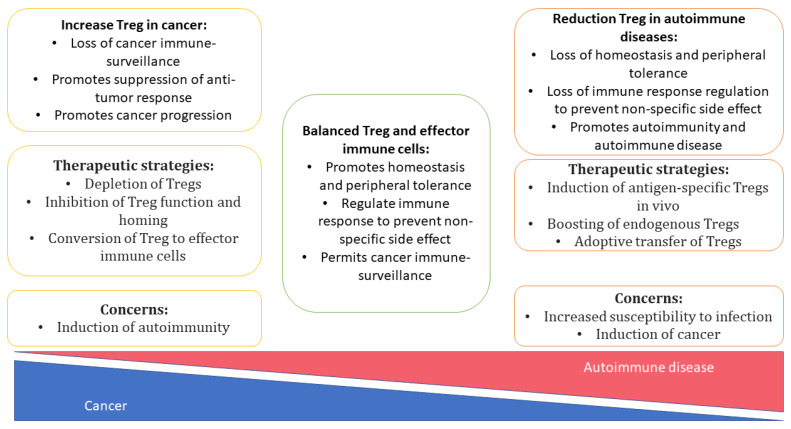
Regulatory T cells in cancer and autoimmunity: role, probably therapeutic approaches and concerns. Treg cells maintenance of immune homeostasis. Imbalance Treg cells have been closely linked to the development of diverse pathologies including autoimmunity and cancer. Treg cells have therapeutic potential either by depletion/inhibition of the Treg cells function to promote anti-tumor immunity, or re-enforcing the function of Treg cells to attenuate chronic/autoimmune inflammation.

In clinical practice, systemic immunosuppressive therapy is used to alleviate autoimmune diseases and pathological reactions to a transplant, e.g., graft-versus-host disease (GvHD). Systemic administration of glucocorticosteroids has many adverse effects, including various opportunistic infectious and even tumors (Figure 3) [142].

Much hope is placed in cell-based therapies involving in vitro-induced Tregs. For instance, polyclonal in vitro-generated FoxP3^+^ Treg cells can slow disease progression in patients with early-stage type 1 diabetes mellitus [143], autoimmune hepatitis [144], systemic lupus erythematosus [145], or inflammatory bowel diseases [146]. Mouse models have yielded promising results in terms of delaying GvHD [80] or solid-organ rejection [147], and even in terms of miscarriage prevention [138]. In clinical trials, despite a suboptimal dose and limited survival of allogeneic Treg cells, investigators registered a somewhat delayed onset of GvHD as compared to a historical control [148]. Additionally, amelioration of chronic GvHD has been demonstrated after Treg cell infusion [149,150] On the other hand, various in vitro-generated Tregs have only a limited survival capacity in vivo [146,148] because Tregs do not acquire epigenetic changes (DNA demethylation and/or histone modifications) [97,151] and transcriptional alterations [152,153] that are characteristic of the body’s natural Tregs, thereby resulting in unstable FoxP3 expression and a weak suppressor function, especially under proinflammatory conditions and after re-exposure [154,155]. Besides, the difficulty is the search for target antigens because antigen-specific Tregs have a more pronounced suppressor potential than do polyclonal Treg cells [80,81,156]. These problems can be solved via the induction of the mTreg phenotype in T cells. Antigenic specificity of mTregs should reduce the severity and the incidence of adverse reactions associated with systemic immunosuppression and with nonspecific Tregs [157]. Owing to the general ability of memory cells to survive and maintain immunity in the body for a long time, we can hypothesize that in vitro–generated mTreg cells can maintain antigen-specific immunosuppression long-term.

To generate Treg cells in vitro, T-cell receptor (TCR) is activated in naïve T-lymphocytes in the presence of TGF-β and IL-2 [158]. Possible conversion of effector or memory T cells into suppressor Tregs may be a promising strategy for the immunotherapy of autoimmune reactions and GvHD. If such an approach is implemented, not only undesirable auto- and allo-specific clones will be eliminated, but also there will be a higher probability of the formation of Tregs having the intended antigenic specificity and memory cell phenotype. Thanks to modern advances in immunology and cell biotechnology, it is now possible to create antigen-specific Treg cells by means of chimeric-antigen-receptor technologies [159]. Such chimeric-antigen-receptor Treg cells show functional stability, good suppressive properties, and long-term in vivo survival, which also reflects specific functional features of memory cells.

It should be underscored that during the development of techniques for the in vivo induction of mTreg cells or their adoptive transfer, it is important to remember that excessive Treg activity can promote tumorigenesis. It is the shift of the immune balance toward Treg cells that can contribute to immune evasion of a tumor via suppression of an antitumor T-cells response [160], and this imbalance correlates with a poor prognosis in some types of cancer [161,162,163]. Tumor-infiltrating Treg cells are phenotypically and functionally different from circulating Tregs [164,165]. For example, genes *CTLA4*, *GITR*, and *CCR4* are expressed in both Treg populations, but the expression is higher in the tumor-infiltrating Treg population. Other genes (*CCR8*, *FCRL3*, and *IL1R2*) are expressed exclusively in tumor-infiltrating Tregs (not expressed in their peripheral counterparts). PD-1, TIM-3, LAG-3, and CD39 are also significantly overexpressed in tumor-infiltrating Tregs [166,167], and they mediate the strong suppressor activity of Tregs. Taken together, these data suggest that such tumor-infiltrating Treg cells may be a subpopulation of tissue-resident mTreg cells. If so, then obtaining this stable phenotype of mTreg cells may be a promising Treg-based strategy for the immunotherapy of autoimmune reactions and GvHD.

Thus, the search for a stable phenotype of mTreg cells and for ways to induce such a phenotype in T cells in vitro is a promising avenue of research at present. In the future, these scientific advances may enable the creation of antigen-specific cell-based vaccines for suppressing GvHD and pathological autoimmune reactions.

## 7. Conclusions

Because of the critical role of Treg cells in inflammation suppression, the identification of cellular and molecular pathways involved in the activation and the maintenance of Treg cells—especially in the formation of regulatory memory—is of paramount importance. Despite the inconsistency of research results on mTreg cells, the phenomenon of regulatory memory undoubtedly exists. The available evidence opens up new possibilities for the isolation of (and studies on the functions of) various subpopulations of memory T cells in a normal state and in inflammatory conditions. The identification of more reliable or more convincing markers of the mTreg function is relevant and important to fully elucidate the roles of mTregs in various human pathological and physiological conditions. There is no doubt that the discovery of ways to induce or to maintain regulatory memory will pave the way to new therapeutic options for autoimmune diseases, and it will facilitate the development of strategies against GvHD. Tolerogenic memory should enable the formation of antigen-specific memory toward a given antigen without causing systemic immunosuppressive reactions.

## Data Availability

Not applicable.
